# Sleep in the Aging Brain

**DOI:** 10.3390/brainsci11020229

**Published:** 2021-02-12

**Authors:** Maurizio Gorgoni, Luigi De Gennaro

**Affiliations:** 1 Department of Psychology, Sapienza University of Rome, 00185 Rome, Italy; maurizio.gorgoni@uniroma1.it; 2 IRCCS Fondazione Santa Lucia, 00179 Rome, Italy

**Keywords:** sleep, aging, cognitive decline, Alzheimer’s disease, EEG, sleepiness, insomnia, obstructive sleep apnea, sleep oscillations, health care

## Abstract

We have entered an era of a steep increase in the absolute and relative number of older people. This well-come phenomenon represents a major challenge for health care. However, maturational changes in sleep associated with aging do not easily appear as main factors, even though sleep alterations in the aging process lead to many detrimental consequences. In this editorial paper, we summarize the present knowledge about the main aging-related sleep modifications and their relevance for health problems and cognitive decline. Then, we present the papers published in the Special Issue “Disturbances of Sleep Among Older People”.

We have entered an era of a steep increase in the absolute and relative number of older people. This welcome phenomenon represents a major challenge for health care, which has to sustain the ability to stay healthy at any age. A healthy status also includes keeping high cognitive and physical functioning, and avoiding or at least minimizing disease and disability.

Among the targets that may be of interest, the maturational changes of sleep associated with aging do not easily appear as a main factor. Still, abnormalities in this maturational process lead to many consequences, such as sleepiness, cognitive impairment, and several cardiovascular events, thus spreading disabilities all over the body. From this viewpoint, exploring the links between normal and pathological modifications of sleep and the brain appears to be of utmost importance to the promotion of successful aging.

Large changes in sleep pattern characterize the elderly population. Age-related modifications in the sleep architecture mainly include advanced sleep timing (i.e., anticipation of both night sleep onset and morning awakening), longer sleep latency, shorter sleep duration, reduced sleep efficiency (i.e., the ratio of time spent asleep to time spent in bed), decreased ability to maintain sleep (i.e., greater sleep fragmentation), increased time spent awake after sleep onset, and reduction of deeper non-REM (NREM) sleep and (to a lesser extent) REM sleep (for a review, see [1,2]). Moreover, the circadian rhythms and the sleep homeostatic process appear less robust with aging [2]. Daytime napping is more frequent in the elderly [3,4,5], and a large proportion of older adults experience excessive daytime sleepiness [3,6,7] albeit several studies suggest a reduced vulnerability to sleep pressure in older subjects [8,9,10].

The electrophysiology of sleep is also affected by age at a microstructural level. In particular, the strongest age-related modifications can be observed in NREM sleep hallmarks, with reduced density and amplitude of slow waves [11,12,13,14], K-complexes [15,16,17,18] and sleep spindles [19,20,21]. Phase-locked synchrony between slow waves and sleep spindles is also affected by age [22,23]. Many primary sleep disorders like insomnia, restless leg syndrome, REM behavior disorder and sleep-disordered breathing are more frequent in older adults [24,25].

Overall, many indices of impaired quality and quantity of sleep characterize aging. Moreover, it is worth noting that different factors associated with aging can have a negative impact on sleep in the elderly, like medical and psychiatric disorders, or environmental, social and lifestyle modification [2].

Such substantial age-related deterioration of the sleep pattern should be considered in light of the association between sleep problems and health. Indeed, many studies found a relationship between sleep duration and/or quality and several health problems like adiposity/obesity [26,27,28,29], diabetes [30,31,32,33], cardiovascular disease [34,35,36,37,38], and mortality [39,40]. A recent meta-analysis found a relationship between short sleep and mortality outcome, diabetes, cardiovascular disease, coronary heart disease and obesity [41]. More recently, another meta-analysis showed that difficulty in the falling-asleep process and nonrestorative sleep were associated with increased risk of mortality, and particularly with cardiovascular disease mortality [42]. Moreover, such a relationship was restricted to the middle- to older-aged population [42]. Starting from these findings, research on the link between sleep and health in the elderly appears essential at a clinical level: wider attention to sleep habits and problems in older subjects may help prevent many health diseases.

The present literature also points to the role of age-related sleep deterioration in cognitive decline [1]. Sleep is essential for memory processes and plastic mechanisms, with a crucial role of different sleep electrophysiological hallmarks like slow-wave activity (SWA) and sleep spindles [43]. Several studies suggest a reduced association between slow-wave sleep and memory in older adults [44,45]. Moreover, the age-related impairment of NREM SWA in older subjects predicts decreased overnight sleep-dependent memory consolidation [14,46], in relation to medial prefrontal cortex grey-matter atrophy [14]. Similarly, the reduction of prefrontal sleep spindles in older adults explains the degree of impaired episodic memory [20]. Age-related deterioration of white matter fiber tracts is associated with reduced sleep spindles, and the level of deterioration predicts whether sleep spindles can promote motor memory consolidation [47]. Crucially, a growing body of evidence suggests that sleep disruption may represent a risk factor for Alzheimer’s disease (AD) [48,49]. AD, the most common age-related neurodegenerative disorder, is characterized by further pervasive sleep impairment compared to healthy aging, with marked alterations of sleep architecture and sleep-wake cycle [50]. The preclinical stage of AD, called Mild Cognitive Impairment (MCI), is also associated with stronger sleep disruption than normal aging [51]. Moreover, several signs of altered NREM and REM sleep electrophysiology in AD and MCI have been observed [48,52,53,54,55,56,57,58,59]. While sleep alterations have often been considered merely a consequence of AD, recent findings point to a bidirectional relation between sleep and AD [48,60,61]. Indeed, sleep disruption is associated with AD biomarkers like β-amyloid and phosphorylated tau in humans [58,62,63,64,65,66,67,68] and animals [69,70,71]. Moreover, β-amyloid levels increase with time spent awake in mice, while the clearance of β-amyloid is predicted by NREM sleep [72,73]. Sleep deprivation and selective SWS disruption in humans induce an increase of the cerebrospinal fluid levels of β-amyloid [74,75,76]. Furthermore, the sleep-wake cycle modulates interstitial fluid levels of tau, and sleep deprivation increases the cerebrospinal and interstitial fluid level of tau and tau spreading [77]. At a longitudinal level, sleep disruption in healthy older humans is associated with AD pathology-related outcomes [78,79,80,81,82], and the proportion of NREM SWA <1 Hz and sleep efficiency selectively predict the following β-amyloid deposition over time [83]. Finally, sleep disruption induces systemic inflammation [61], which is often considered an early event in the AD pathology [84,85]. Overall, the present literature suggests that sleep alterations represent both a risk factor and a marker of AD, raising the possibility that sleep assessment and management may be considered essential for AD prevention, diagnosis and treatment [48,60].

The present collection of articles introduces some critical topics associated with sleep and aging. Bartolacci et al. [86] investigated the influence of sleep quality, vigilance, and sleepiness on driving-related cognitive abilities in older people to identify how sleepiness and sleep quality predict their driving-related cognitive skills. While results confirm some maturational changes of aging (i.e., lower sleep efficiency and lower performance in attention and perception tests), these changes do not necessarily imply a worsened driving ability. In fact, older adults show poorer attentional performance and perception skills in driving tasks while accepting minor risk than younger subjects.

A large study in patients with obstructive sleep apnoea syndrome (OSAS) [87] also debunks another common opinion and shows that OSAS may not necessarily be linked to morning headaches (MH). MH has been considered to be a symptom of OSAS for more than a century. Still, this study suggests that most clinical measures of OSAS parameters are not significantly associated with the probability of MH.

Moving toward a public health perspective with specific attention devoted to healthy lifestyle, another study in a large sample of older individuals living in the insular Mediterranean region [88] shows that midday napping is associated with higher levels of successful aging. Along the same vein, 8.5 h of sleep per day in total, not necessarily slept all together, are associated with the best successful aging level. Another article [89] investigated social and health determinants of insomnia among economically disadvantaged African-American older adults. This study’s merit consists of showing that financial difficulty, smoking, pain intensity, depression, and a higher number of chronic diseases predict insomnia symptom frequency and are associated with higher odds of possible clinical insomnia.

Although the state of the art of existing knowledge should be considered very preliminary, the last article of this collection [90] critically introduces promising techniques to modulate specific sleep characteristics (mostly slow oscillations) with the aim to improve sleep and induce cognitive benefits. This narrative review suggests that techniques with minimal invasiveness, like auditory stimulations delivered during sleep, may be capable of modulating sleep electrophysiology in the elderly population without impacting sleep architecture and the subjective quality of sleep. Although pioneering and very preliminary, these promising studies point to the feasibility and effectiveness of using closed-loop auditory stimulation systems in older people. This approach’s relevance is even greater in light of the mounting evidence on the role of specific sleep changes in the preclinical stage of AD in predicting the onset of cognitive decline [60]. According to this view, the development of intervention strategies and specific techniques effective in modulating sleep electrophysiology may reduce risk factors for AD. This novel view fits with the general notion that some brain plasticity-dependent processes could be improved managing sleep quality, while monitoring EEG during sleep may help to explain how specific rehabilitative paradigms work [91].

We hope that these findings will stimulate interest for further basic and clinical investigations on the role of sleep in healthy and pathological aging, enhancing our knowledge on this research topic.
